# Supplementary material for: All-at-once RNA folding with 3D motif prediction framed by evolutionary information
Source: Nat Methods. 2025 Oct 3;22(10):2094–106. doi: 10.1038/s41592-025-02833-w (PMC12510878; doi:10.1038/s41592-025-02833-w)
Supplement: Supplementary file 3 — Source code to run CaCoFold-R3D. Data and results. [file 41592_2025_2833_MOESM3_ESM.gz › supplemental_material/results/CaCoFold-R3D/CaCoFold-R3D_Rfam/RF01121_sR38.R2R.sto.pdf]

RF01121_sR38

5′-R ● A G G C G A U G A U U U G A C U C C G G A A A A ● C U G A ● Y G A U G A U G A A G U C C A G C C C G A A U G A G C
